# Sacred psychiatry in ancient Greece

**DOI:** 10.1186/1744-859X-13-11

**Published:** 2014-04-12

**Authors:** Georgios Tzeferakos, Athanasios Douzenis

**Affiliations:** 1 Second Department of Psychiatry, University of Athens, Attikon Hospital, 1 Rimini str., Athens 12462, Greece

**Keywords:** Ancient Greece, Animism, Sacred psychiatry, Homer, Tragedians

## Abstract

From the ancient times, there are three basic approaches for the interpretation of the different psychic phenomena: the organic, the psychological, and the sacred approach. The sacred approach forms the primordial foundation for any psychopathological development, innate to the prelogical human mind. Until the second millennium B.C., the Great Mother ruled the Universe and shamans cured the different mental disorders. But, around 1500 B.C., the predominance of the Hellenic civilization over the Pelasgic brought great changes in the theological and psychopathological fields. The Hellenes eliminated the cult of the Great Mother and worshiped Dias, a male deity, the father of gods and humans. With the Father's help and divinatory powers, the warrior-hero made diagnoses and found the right therapies for mental illness; in this way, sacerdotal psychiatry was born.

## Introduction

Three basic trends in psychiatric thought can be traced back to earliest times: (a) organic approach, the attempt to explain diseases of the mind in physical terms; (b) psychological approach, the attempt to find a psychological explanation for mental disturbances; and (c) sacred or magical approach, which can be further divided into the animistic, mythological and demonological models [1]. The origin of the word ‘magic’ leads us back to the Persian religion. The prophet Zoroaster (sixth century B.C.) helped man in his struggle against evil. Aiding Zoroaster in his proselytization of the right road were the priests known as Mah (pronounced Mag), which meant ‘the greatest ones’. In subsequent years, the great Magi lost their high reputation and became known as charlatans and tricksters, hence, the connotation to the word ‘magic’ [2].

The magical sacred approach forms the primordial foundation for any psychopathological development because it reflects a modality of interpretation of reality that is innate to the prelogical human mind. The animistic model is based on prelogical, emotional reasoning, originating from certain historical conditions. Primitive man lived in deep communion with nature and perceived all phenomena to be connected by mysterious forces. Chance does not exist, and everything that happens has a precise meaning because the world is inhabited by animated entities that support every single event. Different feelings and emotions, psycho-sensorial disturbances, and delusions are the work of obscure and ineffable forces that people the world of nature and can act on a man's mind and soul [3].

Greek thought in the middle of the second millennium B.C. transformed the animistic conception into a naturalistic, anthropomorphic theology, in which indistinct and fluid forces were materialized in myths. Every symptom was thought to be caused by a certain deity, which could, if implored, benevolently cure it. The human passions, the emotional suffering from endopsychic conflicts, and the different psychiatric symptoms were projected and concentrated in a divine symbol. The myth was a form of knowledge that took place by symbolizing in concrete divine shapes the phenomena of nature and the complex life of the soul and mind. The ‘anthropomorphism’ of the Greek mythology, where even gods have feelings and emotions, is a historical breakthrough [4].

The genesis and the evolvement of the more elaborate mythological comprehension of the man and the universe are in a direct relationship with the historical dynamic process of an increasing complexity of the social structure. During the pre-Homeric era, in the Minoan and the Mycenaean societies, citizens were divided according to their financial status, to their position in the administrative hierarchy, and to their relationship with the kings. The king in these primitive societies was the sole judicial, legislative, and administrative authority. Another important criterion for the social position and integration in the different groups and ‘fratrias’, in the pre-Homeric and Homeric societies, was the genealogical lineage. Noble families claiming to have descended from mythological heroes gained social power and prestige.

The extensive colonization of the Mediterranean coast by the Greeks led to the emergence of new social groups. The merchants gained wealth and power and also became the bearers of new cultural, scientific, and political ideas taken from the neighboring nations. Gradually and through turbulent strife, the mainstay of the social structure in the ancient Greek world, the city-state (‘polis’) became the cradle of democracy. In the classical era, although the old noble families still held much of their power, the new wealthy aristocracy and the middle social clashes gained access to legislative, administrative, and judicial institutions. This social ‘democratization’ allowed and supported the great scientific and cultural changes that took place in this historical period.

### Shamanism

Primitive man cured his minor troubles through various intuitive, empirical techniques. The first attempts to explain illness were equally intuitive: sometimes simple phenomena having a cause-and-effect relationship were easily understood (overeating and drinking may cause discomfort and thus purgatives will cure it); but that was not always the case.

When the causes of an ailment were not obvious, primitive man ascribed them to the malignant influences of either other humans or divine beings and dealt with the former by magic or sorcery and the latter through magico-religious practices.

In these primitive societies, the typical witch, doctor, or shaman was a person capable of transcending into an ecstatic state, with the help of aromatic herbs, alcohol, seeds, and music. While being in ecstasy, he was able to communicate with the pathogenic spirits, drive them away, and thus cure the patient. In hunting societies, shamans acquire their healing powers from animal spirits [5]. A shaman was able to communicate with the beasts, travel through time and space, sink deep into the world of the spirits, and change his psychic and physical form.

When the Greeks colonized the Black Sea, during the seventh century B.C., they came in contact with shamanic rituals and beliefs. A key figure of shamanism was Pythagoras (sixth century B.C.). He was, in today's terms, a mathematician, astronomer, psychologist, psychiatrist, physician, musicologist, mystic, and philosopher. He gathered excessive wisdom by living through 10 or 20 human generations [6], and he believed in reincarnation (‘metempsychosis’). Through an indefinite cycle of psychic reincarnations, one could achieve immortality, a privilege seized only by the gods. Pythagoras could be considered the ‘father’ of Psychology since, as Porphyrios says, ‘He was the first one to define with precision the anthropocentric science, which teaches us the nature of an individual’ [7]. He was the founder of the encephalocentric doctrine which considered the brain as the seat of human consciousness, sensation, and knowledge and claimed that the psychic organ has a tripartite division, closely resembling the structural theory of Freud: (1) reason, which was the innate category of truth, (2) intelligence, which carried out the synthesis of sensory sensations, and (3) impulse, which derived from the soma. The rational part had its seat in the brain and the irrational one in the heart. Pythagoras considered the mental life to be a harmony supported by the relationship between antithetical forms: love-hate, good-bad, etc. Life itself was regulated according to this theory by opposite rhythmic movements, e.g., sleep-wakefulness, and mental symptoms originated from a disequilibrium of this basic harmony. The work of Pythagoras and Empedocles, originator of the cosmogenic theory of the four classical elements (fire, earth, air, and water), formed the basis for the humoral theory of Hippocrates [8]. Pythagoras stressed the value of group psychotherapy, medical herbs (opium for anxiety, cauliflower and scilla against depression, anis against epilepsy), and music for the treatment of emotionally ill patients [9]. On the other hand, the Pythagoreans avoided cauterizations and incisions [10]. According to Edebstein [11], the ‘Hippocratic oath’ is of Pythagorean origin because some of its main principles are the rejection of assisted suicide and abortion, the prohibition of surgical procedures, and public disclosure of medical cases. Hippocrates and his followers performed surgeries, administered drugs for abortion, and publicly discussed case reports, with direct reference to the patients' names [12].

The psychic immortality was a common belief between the Pythagoreans and the Orphic religious cult, which, according to Herodotus, originated from the Egyptian religion [13]. Fundamental feature of Orphism was the psychic ‘catharsis’ or cleansing, from somatic impulses and passions, through shamanic-like rituals, music, strict dietary practices, and exorcisms [14]. Psychically depressed patients were stimulated with Phrygian music, while the excited ones were sedated with the Doric tonalities. Orphic mysteries mainly practiced in Thrace descended to the Hellenic world from Northern Europe and Siberia [15].

### Between legend and history: Melampus and Asclepius

Ancient Greek medicine was a complex practice perceived as something between myth and reality, as an expression of a magical divinatory, hieratic, and empirical technical practice. Examples of such interrelationship are the myths of Melampus, a priest-psychiatrist who allegedly lived in Argos 200 years before the Trojan War [16], and Asclepius who was considered to be the ‘god of medicine’ by the ancient Greeks [17].

Until the second millennium B.C., a female deity governed the Universe, according to the archaic Pelasgic religion. She was the Great Mother Earth, named Cybele or Selene or Hecate, who dominated man and predated other deities. She gave birth to all things, fertilized not by any male opposite but by symbolic seeds in the form of the wind, beans, or insects [18]. The Great Mother regulated the sexual and affective life and if angered could unleash malevolent influences that brought about zoopathic psychoses. The place of origin of her following is uncertain, but it is thought that she had popular followings in Thrace. Her most important sanctuary was Lagina, a theocratic city-state in which the goddess was served by eunuchs, called Dactyls [19].

The predominance of the Hellenic civilization, in the second millennium B.C., over the Pelasgic, modified the psychopathological interpretation. This fundamental change almost led to a civil war, with lots of killings, especially in the Delphi temple. The Hellenes eliminated the cult of Hecate and worshiped Dias, a male deity, the father of gods and humans. The warrior cult of the hero replaced that of the Mother earth. It was the hero who set himself up as a physician and priest against the forces of evil. With the Father's help and divinatory powers, he made diagnoses and found the right therapies for mental illness; in this way, sacerdotal psychiatry was born around 1500 B.C. [1].

### Melampus

The first and most famous priest-psychiatrist of this new civilization was Melampus, whose existence stands between myth and reality. In Odyssey (χι 223-42) [20], Homer describes the lineage of Theoclymenus, ‘a prophet, sprung from Melampus' line of seers’. This narrative concerning Melampus is done with such brevity that its details must have been familiar to Homer's audience. According to Apollodorus [21], Melampus was the mythological son of Amythaon and Eidomeni and he was born in Pylos around 1400 B.C. about 200 years before the Trojan War. The name Amythaon implies the ‘ineffable’ or ‘unspeakably great’; thus, Melampus and his heirs were the Amythaides of the ‘House of Amythaon’. According to Herodotus and Clement of Alexandria, he was the introducer of the worship of Dionysus who asserted that his power as a seer and healer was derived from the Egyptians [13,22]. Roccatagliata [1], on the other hand, claims that Melampus was the son of God Dionysus, and his father gave him divinatory powers and psychiatric diagnostic and therapeutic abilities. The name Melampus derives from his black feet: just after his birth, he was left in the shade of a tree, but his feet stuck out and were burned by the sun (‘melas’ which means black and ‘pous’ which means foot) [23]. According to the myth, Melampus could understand the language of the animals. This charisma was given with gratitude by two snakes, which were rescued by him, by licking his ears while he was asleep. The cleaning of Melampus' ears by the snakes is considered to be the origin of the use of the snake as a medical symbol, although Asclepius was later associated with this symbol. The father of Asclepius himself, God Apollo, taught Melampus medicine and purified him at the river Alpheios, the waters of which were believed to have curative powers [24].

Melampus became famous for curing Iphiclus, the son of Phylacus. Iphiclus had become impotent, which was a very serious symptom in a civilization that so exalted the phallus and the icon of the dominant male. Melampus used his unique ability to understand the language of the beasts. He sacrificed an ox and observed the eagles that gathered around. The big birds revealed the cause and the cure of Iphiclus' impotence: the last time Phylacus had made a sacrifice, Iphiclus had been terrified of the enormous bloody knife that his father used. In order to calm the boy down, the king had tossed the knife aside. It had hit a tree and become stuck in it, injuring a hamadryad (nymph bonded to a particular tree). The hamadryad cursed the prince with the sickness. In order for Iphiclus to be cured, the knife had to be removed from the trunk of the tree and heated in boiling wine, which the patient should then drink. Melampus found the knife, scraped off the rust, mixed it with wine and gave the mix to Iphiclus to drink for 10 days. After this treatment, Iphiclus was able to have a son, called Podacres.

It has been argued that the rust could have strengthened Iphiclus physically, but one cannot help comparing Melampus' work to the present psychoanalytic therapeutic methods. The healer inquired about Iphiclus' past, the beloved but castrated son, thus revealing the cause of the symptom. Recalling the trauma and resolving the pathological complexes is the next essential step in the psychoanalytic healing process [25].

After the invitation of king Proetus, Melampus cured the women of Argos, whom God Dionysus had turned mad. The daughters of King Proetus, Lysippa, Iphice, and Iphianassa, together with several other young virgins, refused to adore Dionysus, wandered on the mountains behaving strangely and hallucinating: they thought they had become cows. He allowed them to dance and scream, believing that in this way, the women's frenzy would defuse [26]. He then purged the young women with the extract of hellebore roots, a plant containing alkaloids, in order to treat their delusions and made sure they had a bath in river waters. Hellebor can induce massive foul-smelling black stools and can cause convulsions, and it was used to treat different psychic disorders. The ancient Greek word Hellebor origins from the two words ‘ellò’ (‘fawn’) and ‘bibròskein’ (‘eaten’), thus meaning ‘plant eaten by the fawns’ [27].

### Asclepius

The historical facts of Asclepius' life are shrouded in the mists of time. According to Homer, his sons, Machaon and Podalirios, have participated in the Trojan War as heroic fighters and healers. This would place Asclepius' life at about 1300 B.C., a time of waning Mycenaean dominance in the Aegean Archipelagos [28].

Asclepius may have been a historical figure who in successive transmissions of oral tradition became first a hero and then a demigod. According to the myth, Asclepius was the son of the God Apollo and the nymph Coronis. Coronis was the daughter of Phlegyas, king of the Lapiths, who lived in Thessaly. The Lapiths were famous for their struggle with and victory over the Centaurs. This story represents the triumph of the forces of reason and civilization over man's bestial nature. Significantly, after the death of his mother, Asclepius was raised by Cheiron, one of the few surviving Centaurs.

Asclepius' father left a snow white crow to watch over his lover. But Coronis betrayed Apollo in favor of Ischys, with whom she was enamorated. When the crow reported the mortal's transgression, the God cursed it and turned it black. The unfaithful wife was executed by the Goddess Artemis, twin sister of Apollo, and Asclepius was born from the dead body of his mother. As she lay on the funeral pyre, Apollo had a change of heart and turned to Hermes, who cut the still living child from his dead mother's womb. This is considered to be the first Caesarean birth delivered from a dead mother [29]. The name Coronis derives from the ancient Greek word ‘corònos’, meaning crows, considered as being related to an ancient diagnostic and therapeutic ritual. During this practice, it was possible to attend to the incarnation of heroes as ravens or snakes by the intervention of the Goddess Athena.

Apollo took the child to the cave of Chiron, the centaur, to be raised. Chiron was a son of Kronos, half man and half horse, who lived on the mountain Pelion. In his body, he joins together both human and animal realms. The human realm is apollonian: abstract, lucid, and filled with light and reason. The animal realm is chthonic: dark, muscular, virile, and connected to the earth as to sweat and semen. He was viewed as a dark god, and his cave was an entrance to the underworld. Chiron embodies the dual nature of healing: the Apollonian intellect that orders and illuminates through language, reason, and precision and the visceral intuition and animal intelligence which works with torn and bloody flesh. It represents the combination of ‘praxis’ and ‘logos’, treating with the hands and with words. ‘Chiron’ comes from the Greek root meaning ‘working by hand’, which also gives us the English word ‘surgeon’. In Greek religion, hands (‘cheires’) and divine powers were equated [30]. Statues of Asclepius and other Greek healing figures were often gilded just on their hands and fingers [31]. Chiron had been struck by an arrow, meant for someone else, and suffered an incurable wound from which he drew his healing power. With few exceptions, the Greek myths portray the healer with his own persistent wounds. In mythic thought, healing power and woundedness are inseparable. Chiron taught the arts of medicine, hunting, and music to Asclepius, but he was not the only one who helped him become a great healer.

Athena gave Asclepius two vials of blood from the Gorgon Medusa's jugular veins. With the blood drawn from her right side, he could raise the dead and with the blood from the left side he could destroy life instantly [32]. This blood is a perfect example of the concept of ‘phármacon’. This Greek word means a medicine or venom, a substance that could lead to the patient's death or, if given by the proper healer or ‘iatròs’, could save him [33].

Asclepius was depicted in statues as either young or old but often accompanied by a staff with a single serpent twined around it. The snake is a natural symbol of renewal because it sheds its skin periodically to rejuvenate itself. Beneath the temple were labyrinths where live snakes were kept. The name of Asclepius itself originates from the Greek word ‘askalabos’, which means snake.

Asclepius' triumphs led to resentment on the part of the chthonian Hades, the god of the underworld. He resented the loss of subjects resulting from Asclepius' therapies. Hades complained to Dias, who became fearful that the natural order of things would be disturbed. So when Asclepius used Medusas' blood to resurrect a dead man, Dias struck him dead with a thunderbolt [34]. Later, the father of the gods restored Asclepius to life and placed him in the heavens [35].

Cults of Asclepius flourished throughout the Greco-Roman world. Important temples existed at Epidaurus, Athens, Ephesus, Tricca, and Kos [36]; one was erected in Rome on the Tiber Island in 300 B.C. Located in peaceful and attractive settings, these temples were established to encourage patients to believe that there were good reasons to want to recover. The preservation of a climate of spiritual healing was largely entrusted to a priestly group derived from a few selected families. The priests established procedures for worship, suggested appropriate sacrifices at Asclepius’ altar and sought to create a holding environment for the pilgrims.

The relationship between medicine and the temples of Asclepius did not seem to be competitive but rather complementary. The Hippocratic physicians of the island of Kos constituted themselves as a kind of guild under the name of the ‘sons of Asclepius’. The priests were helped by lay practitioners or Asclepiads who supplemented the ministrations of the priests and guided the pilgrims in their healing activities [37]. They tended to focus their attention on the corporeal and pragmatic elements of the treatment program and also acquired a number of specifically medical skills. Vials for medical administration and surgical implements were discovered in the ruins at Epidaurus.

Included among the temples' treatment techniques were a balanced diet, a daily massage, quiet sleep, priestly suggestions, and warm baths, all of which were thought to comfort and soothe patients [38]. Each temple specialized in the treatment of different diseases: in Tricca hysteria, in Epidaurus psychogenic disturbances of consciousness treated with blood from Medusa, and in Megara psychoreactive manifestations caused by different passions. The main therapeutic procedure, though, remained that of therapeutic divination through the interpretation of dreams [39]. After rites of purification, and offerings to Apollo and Asclepius, incubation involved staying within a sacred central region of the temple grounds, the ‘abaton’, often constructed as a labyrinth sunken into the ground (inside the MotherEarth and resembling the underworld). There, the afflicted person slept or tranced to experience healing dreams or visions. The interpretation of these dreams, by the priests of the temple, elucidated and revealed the cause and the proper cure for the disorder [40]. A typical cure is recorded in the temple inscriptions from Epidauros, a man with a urethral stone comes to the god for help. He lies down and has an erotic dream of intercourse with a beautiful man and has an ejaculation that expels the stone [41]. The bestdocumented case of long-term relationship with the God Asclepius in order to heal a variety of illnesses, psychic as well as somatic in origin, is that of Aelius Aristides, an orator and hypochondriac, who lived in the second century A.D. and a contemporary of Galen (who also used incubation as a therapeutic method in the Asclepeion of Ephesus), whom he once consulted. Aelius spent almost a decade wandering from one Asclepian temple to another, in different parts of the Mediterranean world, seeking and obtaining divinely inspired dreams, including some in which he had direct interviews with the god. Incubation or ‘temple-sleep’, as a psycho-therapeutic method, was also used in Ancient Egypt, and it was associated with Imhotep (2655-2600 B.C.), the earliest known physician in history [42]. Imhotep or Imuthes in Greek, meaning ‘he who comes in peace’, besides being a physician, was also an engineer and architect; he was the architect of the step pyramid at Saqqara built by the Pharaoh Zoser. He was worshiped at Memphis, and a temple was constructed in his honor on the island of Philae [43].

In ancient civilizations, dreams were analyzed in order to foresee the future, to understand the soul, and to make political, military, and religious decisions [44]. The oldest historical mention of the importance of dreams dates back to the early Mesopotamian civilizations. Gardiner translated the earliest book of dreams, found in the Chester Beatty Medical Papyrus (around 2000-1790 BC), time of the 12th dynasty, which contains interpretations of all kinds of dreams [45]. In ancient Greece, until the sixth century B.C., the complex oneiric life was considered a premonitory divine sign (Odyssey ω 9–12) [20]. During this period, however, a crucial change took place: the dream, from an important element of the relationship between man and god, became the expression of inner truth, useful not only to divine but also to attain a more profound knowledge of the human soul [46]. In Odyssey (τ 560-7) [21], Homer describes two kinds of dreams: (a) the real and valuable ones, arising from the ‘keratin’ gate (the eyeball), foretelling the future and warning for incoming dangers and (b) the deceitful (Odyssey ζ 21-40) [21], penny, and futile, arising from the ‘elephantine’ gate (the teeth), which could only lead to disasters (Iliad β 5-34) [47].

### Soul and mental health in Homer

According to Homer, Asclepius was the first one to distinguish between medicine and surgery: he gave the power of recovery to his son Podalirius and the ability to treat wounds to Machaon. On the other hand, no separation between psychic and somatic conditions was made [18]. The Homeric poems, most likely composed in the second half of the eighth century B.C.; they are representatives of an oral tradition reflecting the views of Greeks living in former times [48]. Their study is so invaluable because in order to bring a culture into focus, one must not only take into account the so-called scientific data available for the time and place but also one must turn to the poet and playwright, myth-maker, and legend-bearer for they re-create the myths in terms of their own genius and tap the culture at its deepest unconscious level [49].

Τhe Greeks of Homer had not yet developed a unitary concept of the psychic life [50]. Homer distinguished indeed different types of soul. There was a soul not located in a specific area, a kind of ‘life-soul’ or ‘breath-soul’ that animates the body, called ‘psychē’, and different body souls, called ‘thymos’ or ‘noos’ or ‘menos’ [51]. The ‘psychē’ was representative of the individual's life and identity. It was not associated with any specific body part; it lacked any psychological attribute and possessed merely eschatological traits: at the moment of death, it fled away from the limbs or through the wounds and departed to Hades where it began an afterlife (Odyssey λ 566-7, Iliad χ 466-7, χ 160-1, ι 409-10, π 826, π 856) [20,47].

By contrast, the body souls were active during the waking life. The ‘thymos’ was the source of emotions, the potency to set the body in movement. It resided in the chest, concentrated into the ‘phrenes’ (the diaphragm or the lungs) [52], the location of feelings. The ‘noos’ was associated with rational and intellectual attitudes, intervening when the subject had to reason and ponder. It resided in the chest, without any specific anatomical relationship, and it was the prodromal figure of the concept of the mind. The ‘menos’ was the aggressive impulse, the fury, the rage in the battle. It was located in the chest but was not a physical organ.

In Homer's poems, we find also the terms ‘kradiē’ or ‘ētor’, which are translated with ‘heart’. Although Crivellato and Ribatti [48] claim that these terms were not related to intellectual functions but only designated some source of feelings, Vasmatzidis [4] considers the heart (‘kradiē’ or ‘ētor’) to be the main seat of the psychic life, the cognitive center, and the origin of many emotions.

In Homer's Odyssey and Iliad, the mystery of the inner mental life was explained by means of an objectivation and projection. Every irrational, reckless, insane behavior, and every claudication of the mind,the reason was attributed, not to organic or psychological causes but to the action of an invisible fluid sent to individuals by an external demonic force or an angry deity (Odyssey λ 61, φ 237) [20]. Homer believed that mental disease, madness, or insanity, called ‘Até’, had supernatural origin (Odyssey ι 410-11, ψ 11-12, Iliad ζ 234-6) [20,47]. All sorts of mental and physical events were credited to intervention by demons (‘alástor’) or by the gods [39]. In Odyssey, when the old nurse Eurykleia announces to Penelope that Odysseus has returned and has killed the suitors, she tells the old woman that the gods have driven her mad and wrecked her mind, which is ordinarily sane [20]. In Iliad, Agamemnon trying to find excuses for his offending and insulting behavior against Achilles (he took away Achilles' mistress Briseis), which led to Achilles' denial of joining the battle, invokes ‘Até’, sent by Dias and the Erinyes, the night stalkers [47].

Hesiod in his Theogony [53] describes the birth of the Erinyes: Cronos, the son of Uranus and Earth, urged by his mother, castrated his father with a jagged sickle. The blood drops from Uranus' castrated genitals fall on Earth and gave birth to different deities and creatures: the strong Erinyes, the great Giants with gleaming armor and long spears, Nymphs called Meliae, and above all the Goddess Aphrodite.

‘Até’, this divine insanity, conceptualized the idea of punishment, only once in the Homeric poems. In Iliad's ι 502-12 [47], ‘Litaé’, the goddesses of compassion and contrition, sisters of ‘Até’, limp after their vicious sister trying to comfort her victims. But if someone rejects them, they call upon Dias to send against him their avenging sister. Hesiod, in his ‘Works and Days’ presents ‘Até’ as the punishment for committing ‘Hubris’, the arrogant transgression of the universal law of humility and modesty [53]. Later on, ‘Até’, under the influence of the Ionian philosophers and the ethical code of Orphism, is equated to the concept of punishment [4].

### Insanity in the tragedians and Herodotus

The theme of madness as a divine punishment for wicked deeds can be seen in the work of the tragedians Aeschylus, Sophocles, and Euripides and the contemporary of the tragedians, the historian Herodotus. Tragedies contain some of the most vivid descriptions of insanity in ancient literature, so vivid that the writers must have made personal observations of the clinical presentation of insanity [54]. This tragic divine madness is considered to be caused by a god or gods, usually as a punishment for wicked or impious action.

In both Aeschylus' Eumenides [55] and Euripides' Orestes [56], Orestes suffers a psychotic attack: terrifying visions, wild ravings interspersed with periods of sleep, fits of despair, refusal of food and drink, and repeated wishes of death. Orestes had murdered his mother, Clytemnestra, to avenge the death of his father, Agamemnon, who had been slain by her, upon his return from the Trojan Wars. Her accomplice was her lover Aegisthus, Agamemnon's cousin.

Erinyes pursued the perpetrator around the world. With the help of Apollo and Athena, he was brought to trial in Athens with a jury of 12 Athenian citizens. Apollo was a witness for the defense, but he did not seem to make out a strong case. The judges' verdict was equally divided, and it was Athena's vote that tilted the scale in favor of Orestes. This ‘Athena's vote’ closely resembles one of the basic principles of today's judicial system, the principle of ‘in dubio pro reo’. It is also Athena's intervention that finally calms down the Furies-Erinyes by assuring them that the trial has been fair, that they are neither defeated nor disgraced, and that the position offered to them in Athens gives them perpetual honor and usefulness. Thus, the fearful and cruel Erinyes turn to Eumenides (‘kind ones’) and pronounce blessings instead of curses [57].

Another classical description of mental illness is that of Sophocles in his play, Ajax [58]. Among later Greek writers, Ajax was regarded the typical ‘melancholic man’, whose characteristics closely resemble the manic–depressive illness.

The armor of Achilles, after his death, was to be given as a prize. The two candidates for receiving this invaluable prize were Ajax and Odysseus because they were the ones who fought more bravely to retrieve the corpse of the dead warlord. Odysseus, with the aid of Athena, was the recipient. Ajax was very frustrated and under the influence of ‘Até’ sent by Athena (the reason for his madness was due to his ‘hubris’, when he foolishly denied Athena's help in the battle); he decided to murder the leaders of the Greek army in retaliation. Tecmessa, Ajax's wife describes with horror the delusional breakdown of her husband: in his psychotic madness, he slaughters the cattle, binds the beasts and beats them with a singing whip, and ‘pours forth such awful curses as no man, but some demon, must have taught him’. He talks with shadows and mocks the Greeks, laughing at them with irony. It was Zeus' intervention, with a lightning, that put an end to this horrible frenzy.

Ajax recovers and faces the dark reality: he stands ashamed in front of his compatriots, having lost his dignity and their respect. The donation of Achilles' armor to Odysseus marks the end of an era and the beginning of a new one: the ideal icon of the valiant warlord-hero is replaced by the cunning wisdom of Odysseus. The drama climaxes when Ajax commits suicide by falling on his sword.

Heracles falls outside this pattern of punishment for wrong doing. In the Euripidean tragedy ‘Heracles’ [59], the hero suddenly becomes a violent madman, murders his wife and children in his father's palace, under the delusion that they are his enemies and finally sinks into an exhausted sleep. His madness is inflicted upon him by Lyssa, the personification of insanity, on the orders of Hera, Dias' wife. Hera persecuted Hercules throughout his life because she was jealous of Dias' affair with Heracles' mother, Alemene.

Lyssa, gorgon-faced (being able to turn into stone anyone who dared look her in the eyes) and holding a goad, appears in a black chariot on the roof of the palace, accompanied by Iris. Iris is the symbol of the rainbow and a messenger between earth and sky, between humans and gods. Lyssa expresses her doubts and objections; Heracles is not a common or injustice man. Driving him mad and, thus, making him the murderer of his wife and children is a horrifying and terrible act.

The other form of Athenian playwright was comedy, with the most prominent and popular writer being Aristophanes. In one of his comedies, *The Wasps*, he describes the methods used to cure a patient's mental disorder [60]: persuasion with soothing words, washing and purification, incubation in the temple of Asclepius in Aegina, participating in mystic rites (wild Corybantic dance and music), and finally, if all else had failed, constraint in the house. Of these forms of treatment, purification (‘katharmos’) was believed to be a method of freeing a person or a community from a religious pollution (‘miasma’) and is one of the magical treatments for mental disorders. Insanity, therefore, was regarded in popular thought to be caused by some kind of religious pollution, which could often be unintentional [61]. Related to this belief was the ritual of ‘pharmakou’; every year, a community used to pick up a deformed, outcasted, criminal or mentally disturbed individual and exile him in order to avert or minimize catastrophes and disasters. In Sophocles' ‘Oedipus Tyrant’ [62], the patricide and incest Oedipus, after the revelation of his hideous crimes, is expelled from Theba to avoid the ethical pollution of the city and its inhabitants.

The theme of madness as a divine punishment for wicked deeds can also be seen in the work of the contemporary of the tragedians, the historian Herodotus. Among the great and mad figures of sixth and fifth century B.C. history, who appear in Herodotus' narrative, is the Agiad King of Sparta Cleomenes. After being recalled to Sparta, for suspected subversive activities, Cleomenes went mad, although it seems that he suffered from a degree of mental illness throughout his life. At around 50 years of age, he fled Sparta, being seized by ‘fear of his people’ and sought assistance from the Arcadians to attack Sparta. When the Spartans brought him back, his behavior was so disorganized that they had to confide him to the stocks, bound and guarded. There he committed suicide in a particularly revolting and cruel manner: he sliced his flesh into strips with a knife he took from one of his guards. Some Greeks believed that the cause of Cleomenes' madness was the divine punishment for different acts of sacrilege that he performed: he had invaded Eleusis and ravaged the shrine of Demeter and Persephone, he had showed no respect for supplicants in a temple at Argos, and, above all, he had corrupted the Priestess of Apollo at Delphi to lie about the legitimacy of his rival, King Demaratus, which had resulted in Demaratus being driven from his throne. On the other hand, Herodotus believed that Cleomenes' madness was the result of his alcohol abuse.

Herodotus presents another example of madness affecting a King; King Cambyses of Persia (c. 540 B.C.) was a cruel, irrational tyrant, who treated his compatriots with the savagery of a lunatic.He killed a sacred animal, the Apis bull, married two of his sisters concurrently, and then killed his favorite sister/wife and his brother. Many believed that the slain of Apis calf was the reason for his madness, while Herodotus adopted a more mundane explanation: he suffered from congenital epilepsy or ‘sacred disease’ and also he drank alcohol excessively [13].

## Conclusions

Sacred psychiatry differs substantially from superstition and from the psychiatric treatments carried out by shamans and magicians, not only in its organization but also in its therapeutic approach and its coherent interpretive method. It was a part of a complex mythological system, and its representatives were medical-sacerdotal charismatic persons who, at the same time, collected clinical and semiotic data, thus paving the way for Hippocrates, the father of Medicine, to lay the foundations of today's medical practice. Hippocratic medicine rejected the belief for any ‘divine’ intervention to the appearance and development of a disease—without the intention though to doubt the traditional templar religion—and denied any magical therapeutical application that took place with incantations, prayers, and purifications. Hippocrates never denied the divinity of the diseases, but this divinity existed through nature. Nature is the field where man, with the help of his spiritual and intellectual tools, struggles and competes for freedom.

## Competing interests

The authors declare that they have no competing interests.

## Authors' contributions

GT carried out the collection of the data and wrote the article and DA had the overall supervision of the process, wrote part of the article, and reviewed it. Both authors read and approved the final manuscript.
